# Dissecting Cellulitis of the Scalp: Case Discussion, Unique Considerations, and Treatment Options

**Published:** 2014-06-06

**Authors:** Mairin A. Jerome, Donald R. Laub

**Affiliations:** ^a^University of Vermont College of Medicine; ^b^Fletcher Allen Health Care, Burlington, Vt

**Keywords:** dissecting cellulitis of the scalp, scalp excision, treatment, skin grafting, follicular occlusion triad

## DESCRIPTION

A 23-year-old African American man presented with a several-year history of dissecting cellulitis of the scalp refractory to medical management. Two surgeries were performed for debridement and split-thickness skin grafting, ultimately of the entire scalp, followed by negative pressure wound therapy. He showed complete healing at the 4-month follow-up.

## QUESTIONS

1. **What is the clinical presentation and predilection of dissecting cellulitis of the scalp (also known as perifolliculitis capitis abscedens et suffodiens, or Hoffman's disease), and what other clinical implications should be considered?**
2. **Within what group of conditions is the disease classified, and what is its pathophysiology?**
3. **What are the options for management?**
4. **To what depth should the scalp excision extend for curative treatment?**

## DISCUSSION

**Figure 1:**
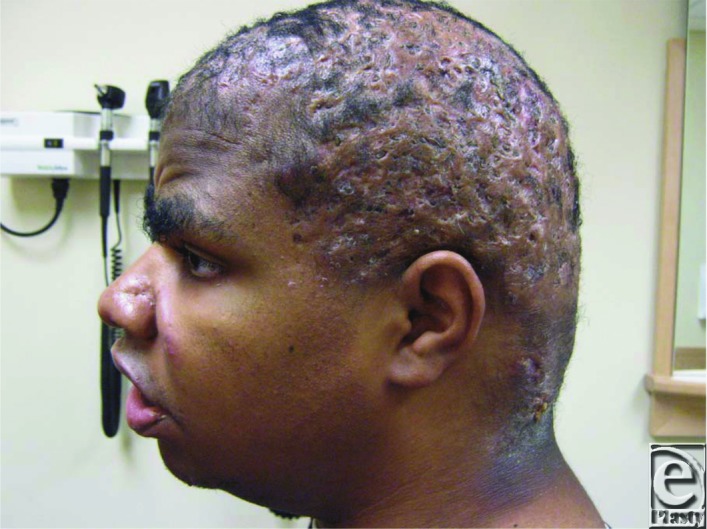
Patient at preoperative consultation. Alopecia, scarring, and pustules visible.

Dissecting cellulitis of the scalp, also known as perifolliculitis or Hoffman's disease, presents clinically as relapsing, suppurative, tender nodules on the scalp that eventually form draining sinuses (Fig 1). This often leads to subsequent scarring and alopecia, which can be both painful and disfiguring.^1^ The condition is very rare and most common in young adult black men in the second to fourth decades of life.^1^ This condition often causes psychological distress due to the cosmetic appearance of the scalp,^3^ as well as possible odor secondary to infection. Other clinical conditions may be associated, including sternoclavicular hyperostosis, polyarticular arthritis, and HLA B27-negative spondyloarthropathy, among others.^3^ Squamous cell carcinoma arising in the setting of dissecting cellulitis of the scalp, though rare, has been described^2^ and should be excluded. This is particularly true in relapsing cases, which also increases risk of osteomyelitis.^3^

**Figure 2:**
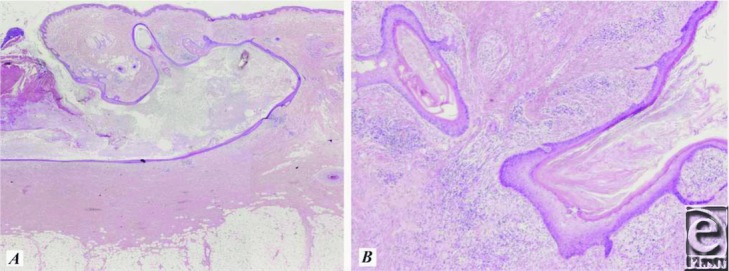
(*a*) Sinus tract at 2x. (*b*) Hyperkeratosis and perifollicular inflammation at 40x.

Dissecting cellulitis of the scalp is one of the 3 conditions identified within the “follicular occlusion triad,” along with supportive and acne conglobata. Although they occur in different areas of the body, these conditions are characterized by folliculitis leading to deep scarring. Each results from pore occlusion due to keratin retention, causing pore dilation, bacterial infection, and sinus tract formation.^4^^-^5 Histologically, dissecting cellulitis of the scalp is characterized in the early phase by a heavy infiltrate of follicular and perifollicular neutrophils, resulting in abscess formation in the dermis of the scalp.^4^ As the condition progresses, draining sinus tracts form, and the inflammation becomes both acute and chronic with varying degrees of follicular destruction (Fig 2). Although bacteria often plays a large role in the pathogenesis of the condition, no specific pathogenic organisms have been associated.^6^

Multiple treatment options have been described, though recommendations are based on small series or case reports due to the dearth of larger clinical trials. Treatment is based largely on severity. In mild cases, first-line treatment consists of improved scalp hygiene, antiseptics, topical antibiotics, lesional aspiration, oral antibiotics, and corticosteroid injections. In more severe cases, oral antibiotics combined with rifampin plus or minus corticosteroids have been shown to be effective. Isotretinoin treatment has resulted in remission if used for 4 months after clinical control is established.^6^ One case report cites complete remission after 6 months of treatment with zinc sulfate without relapse at 5 year follow up.^7^ A few case reports have demonstrated success with anti-TNF alpha therapy, and laser epilation also has a role in treatment before the occurrence of inflammation.^6^

**Figure 3:**
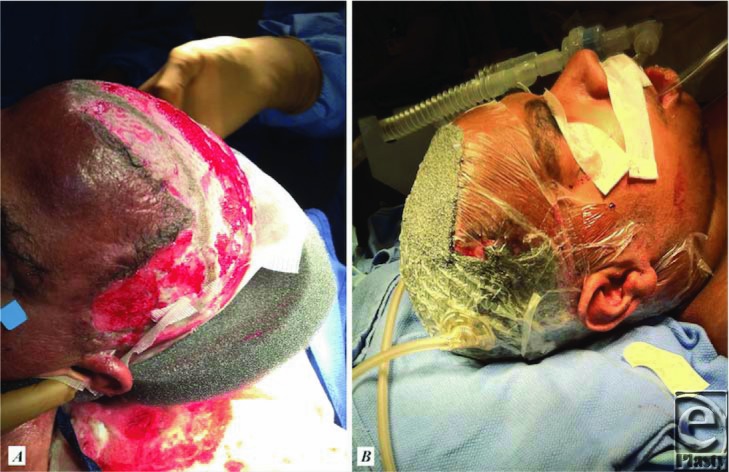
(*a*) Perioperative placement of wound VAC. (*b*) Complete wound VAC placement. VAC indicates vacuum-assisted closure.

**Figure 4:**
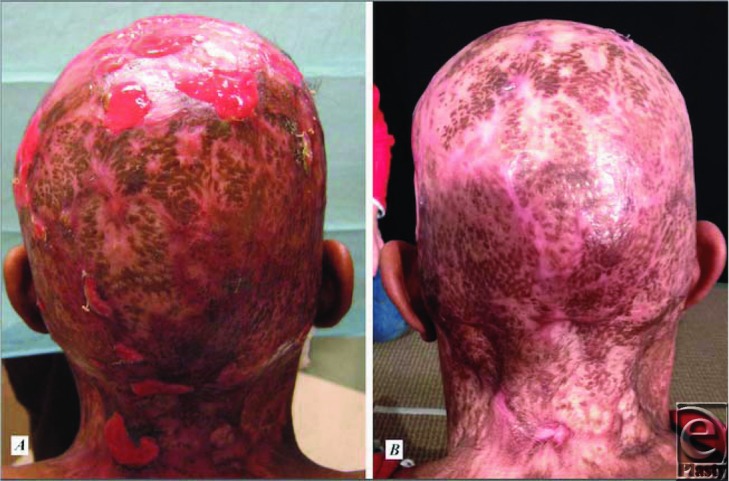
(*a*) At 3 weeks postoperatively. (*b*) At 4 months postoperatively.

For intractable cases, surgical excision and split-thickness skin grafting are often required and successful. Several case studies have reported success in achieving long-term remission and possible cure of the disease with complete scalp excision and split-thickness skin grafting.^2^^,^^5^^,^^8^ The successful surgeries have excised to a depth below the presence of disease, usually to the galea or just subgaleal.^5^^,^^8^ Vacuum-assisted closure dressings (Fig 3) have been used with success^8^ and patients exhibit complete healing (Fig 4) with alopecia at a 9- to 10-month recheck.^5^^,^^8^

In summary, dissecting cellulitis of the scalp is a rare condition primarily affecting young adult African American men with a clinical presentation consisting of tender, suppurative scalp nodules that eventually form sinus tracts as the condition progresses. The severity of disease is variable and treatment should be determined accordingly. The mildest cases can be managed conservatively with drainage and topical treatments, while the most severe and relapsing cases often need surgical excision and skin grafting. It is important in these patients to assess for associated clinical conditions, as well as squamous cell carcinoma and osteomyelitis.
